# Radio Frequency Electromagnetic Fields Exposure Assessment in Indoor Environments: A Review

**DOI:** 10.3390/ijerph16060955

**Published:** 2019-03-17

**Authors:** Emma Chiaramello, Marta Bonato, Serena Fiocchi, Gabriella Tognola, Marta Parazzini, Paolo Ravazzani, Joe Wiart

**Affiliations:** 1 Istituto di Elettronica e di Ingegneria dell’Informazione e delle Telecomunicazioni IEIIT CNR, 20133 Milano, Italy; marta.bonato@ieiit.cnr.it (M.B.); serena.fiocchi@ieiit.cnr.it (S.F.); gabriella.tognola@ieiit.cnr.it (G.T.); marta.parazzini@ieiit.cnr.it (M.P.); paolo.ravazzani@ieiit.cnr.it (P.R.); 2 Dipartimento di Elettronica, Informazione e Bioingegneria DEIB, Politecnico di Milano, 20133 Milano, Italy; 3 Télécom ParisTech, LTCI University Paris Saclay, Chair C2M, 75013 Paris, France; joe.wiart@telecom-paristech.fr

**Keywords:** RF electromagnetic fields, exposure assessment, indoor environments

## Abstract

Exposure to radiofrequency (RF) electromagnetic fields (EMFs) in indoor environments depends on both outdoor sources such as radio, television and mobile phone antennas and indoor sources, such as mobile phones and wireless communications applications. Establishing the levels of exposure could be challenging due to differences in the approaches used in different studies. The goal of this study is to present an overview of the last ten years research efforts about RF EMF exposure in indoor environments, considering different RF-EMF sources found to cause exposure in indoor environments, different indoor environments and different approaches used to assess the exposure. The highest maximum mean levels of the exposure considering the whole RF-EMF frequency band was found in offices (1.14 V/m) and in public transports (0.97 V/m), while the lowest levels of exposure were observed in homes and apartments, with mean values in the range 0.13–0.43 V/m. The contribution of different RF-EMF sources to the total level of exposure was found to show slightly different patterns among the indoor environments, but this finding has to be considered as a time-dependent picture of the continuous evolving exposure to RF-EMF.

## 1. Introduction

The pervasive use of wireless communication devices in each aspect of everyone daily life, emphasized the need for assessing the level of radio-frequency electromagnetic field (RF-EMF) exposure. Several studies aimed to estimate the exposure level for RF-EMF source in the 10 MHz–6 GHz frequency range, related to radio and television broadcasting, base stations for telecommunications and mobile phones, cordless (DECT) phones, RF identification tagging systems, and wireless communications applications, such as WiFi, wireless local area network (WLAN) and mobile telecommunications networks—Global System for Mobile Communications (GSM), Universal Mobile Telecommunications System (UMTS) and Long Term Evolution (LTE) [1]. Different approaches with different levels of accuracy have been used to estimate RF-EMF exposure, e.g., spot and long term measurements, personal measurements with portable exposimeter, simulations based on computational electromagnetics techniques.

Surveys with different aims have been carried out on: population surveys, i.e., studies aimed at investigating the differences between the levels of exposure of different groups of individuals (see, e.g., [2,3]), micro-environmental surveys, i.e., studies aimed at investigating the differences between the exposure in relevant micro-environments, both indoor and outdoor [4], and temporal studies, aimed at investigating temporal variability of the exposure, by measuring along the same itinerary at the same time over a period of several months or years [5,6]. 

Nowadays the rapid evolution of technology and network infrastructures towards the fifth-generation (5G) systems requires a similar evolution in the methods of assessment of the exposure, in order to face the increasing complexity of the spatio-temporal indoor and outdoor exposure. This is particularly needed in indoor environments, in which it was estimated that people spend about 70% of their daily time [7]. In an indoor environment, the RF exposure depends on outdoor sources such as radio, television and/or mobile phone antennas, as well as on indoor sources such as e.g., mobile phone handsets, DECT base stations, Wireless Local Area Network (WLAN) net, etc. While radio and television transmitters have a large coverage area and therefore operate at relatively high power levels, the power level inside a building can be up to 100 times lower than that outside the building, depending on the number of windows and the structure of the walls, and the exposure may vary from floor to floor. As to the indoor sources, their number and their locations, as well as the structure of the rooms and their furniture, are affected by a lack of information especially for private environments. All these aspects cause the assessment of the exposure to RF-EMF to be affected by uncertainties and represents a challenging task.

In this document, we aimed to review research efforts done in the last ten years in assessing RF-EMF exposure in indoor environments, presenting our findings in terms of those RF-EMF sources that were found to cause exposure in indoor environments, in terms of the different indoor environments that have been investigated and in terms of the different approaches used to assess the exposure. A summary of the levels of exposure for each type of indoor environment will be presented, highlighting limitations and gaps. 

## 2. Methods 

### 2.1. Literature Search Strategy and Inclusion Criteria

A literature search of the research results published between 2008 and 2018 was performed using Scopus database. The search terms were derived as a combination of the various ways of describing the exposure characteristics (e.g., radio-frequency electromagnetic fields, RF electromagnetic fields, radio frequency EMF, microwave EMF, etc.), the environment (e.g., indoor, residential, school, offices, etc.) and the exposure assessment (e.g., exposure assessment, exposure measurement, dosimetry, exposure personal measurements, exposure spot measurements, etc.). No specific EMF source was used as a search criteria.

Only peer reviewed articles published in the English language were considered. The eligible studies had to report mean RF-EMF exposure levels for indoor environments, considering the whole exposure range (10 MHz–6 GHz). 

In case of personal measurements in which the levels of exposure were measured both indoor and outdoor, only those studies in which it was possible to clearly separate the values measured indoor from the other values were considered. In case of duplicate publications, we included the article with the most comprehensive data, while in case of publications in which results included measurements described in previous studies, also these previous studies were considered, even if published before the 2008.

### 2.2. Methods Used to Summarized Exposure Levels

In order to make possible to compare the levels of exposure observed in different studies, all the values reported as power density were converted to E-field strength (V/m) by:

(1) $$E\;(V/m)=\sqrt{Z_{0}*Power\;density\;\left(W/m^{2}\right)}$$

where *Z*_0_, equal to 377 Ω, is the impedance of vacuum.

## 3. Results

The results are organized as follows: first, an overview of which RF-EMF sources, indoor environments and assessment strategies were used for evaluating the RF-EMF exposure levels in the various considered studies. In the second part the numerical values of the RF-EMF exposure levels found in different studies were summarized by indoor environment.

### 3.1. Sources

In literature, both sources placed indoor, such as mobile and cordless phones, FM radio, smart TVs, Wi-Fi routers, laptops, tablet computers, and sources placed outside the environment, such as radio and television broadcasting and base stations for telecommunications have been investigated as influential on the level of exposure to RF-EMF (Table 1). 

Actually, the total indoor exposure can be categorized as due to both sources placed indoor, and sources placed at a sufficient distance from the subject, such as all the sources placed outdoor [8]. Indoor sources, such as mobile and cordless phone calls, operate in close proximity to the head or body and account for the majority of RF-EMF although sporadic RF-EMF exposure. Outdoor sources, such as mobile phone base stations, occur from much greater distances away, resulting in a much lower, but practically continuous during the day, level of RF-EMF exposure, depending of the line of sight, vegetation, reflective walls and building materials. If, on one hand, the indoor attenuation of the power level from base stations could result in a lower exposure level, on the other hand any mobile device placed indoor is forced to radiate higher power for the transmitted signal to reach the base station ensuring an acceptable signal quality [8,9]. In the last years femtocells, i.e., miniature base stations specifically designed for the enhancement of the coverage and capacity of a mobile service in indoor environments, have been deployed to overcome the power attenuation from outdoor to indoor. Some studies focused on the assessment of RF-EMF exposure from a femtocell, both considering the exposure due to the femtocell itself [10,11,12] and comparing the received and transmitted signal power of a mobile phone with and without the femtocell [13,14,15]. A study by Aerts et al. [16] compared the RF-EMF exposure of a mobile phone user that is either connected to an Femtocell Base Station (FBS) or a conventional macrocell base station while in an office environment considering both near and far field exposure, finding that, in average macrocell coverage and mobile phone use-time conditions and for UMTS technology, the total exposure can be reduced by a factor 20 to 40 by using a femtocell, mostly due to the significant decrease in the output power of the mobile phone.

As reported in Table 1, most of the sources for which the assessment of the exposure levels accounted for in literature showed working frequencies higher than 100 MHz. No one of the papers considered in this study reported the exposure levels for sources, such as induction cookers, wireless alarm systems or Radio-Frequency Identification devices, with slightly lower working frequency.

### 3.2. Indoor Environments

Many different indoor environments, summarized in Table 2, have been analyzed in order to assess the exposure to different types of RF-EMF sources. Most studies dealing with public environments, which often play the role of workplaces, focused on the exposure in offices, due to the high number of WiFi sources that are usually present [17]. The second most investigated public environment is represented by schools, due to the presence of children of different ages and to the attention paid to their precocity of exposure to RF-EMF [18]. Larger spaces like hospitals/health care facilities, universities and commercial locations (coffee shops, fast food outlets, general merchants), tourist visitor centers and hotel rooms have been investigated by only few studies, based mainly on spot and personal measurements.

Also private places such as houses and flats were investigated, with a particular attention to the bedroom, as suggested by Tomitsch and Dechant [19]. Typical environmental factors influencing the level of exposure are linked to the degree of urbanization of the environment under exam, e.g., rural, suburban, urban [3,20], the percentage of urban ground use of the area and the population density, e.g., the number of social meeting places such as pubs and railway stations, in which more people are present, potentially showing higher exposure [21,22].

Special attention was given to public transportation, such as trains, as several studies on the exposure of the general public to radio-frequency (RF) electromagnetic fields (EMF) have established that public transport (bus, train, etc.) has become the dominant micro-environment in terms of RF-EMF exposure, with the largest RF-EMF strengths attributed to mobile-phone use [20]. This is due to several factors. First, the fast movement of the train, forcing the mobile phone to repeatedly connect to a different base station (macrocell) during which the power of the mobile device is set to its maximum; second, the metal frame of the train that behaves more or less like a Faraday cage, significantly attenuating any signal that penetrates it (hence, any mobile device inside the train is forced to radiate more strongly for the transmitted signal to possess enough power to reach the base station); and third the large amount of people present in a small environment which is the train car, increasing the chances of mobile-phone use, and thus reinforcing the aforementioned factors.

### 3.3. Assessment Strategies

Many different strategies have been used to assess the exposure to RF-EMF sources in indoor environments, both based on measurements and simulations methods. In particular, spot and long-term measurements by exposimeters placed in fixed position for a certain experimental duration were used to obtain information about environmental far field RF-EMF exposure in specific indoor environments. Personal exposure measurements, obtained by using portable exposimeters or personal exposure meters (PEMs), allowed to obtain information about the level of RF-EMF exposure received by an individual across different locations and times. Very specific tools, i.e., mobile phone based instruments such as smartphone based applications (see, e.g., the app XMobiSens used in the pilot study of Goedhart and colleagues [23]), were used in epidemiological studies to obtain information linked to the near field RF-EMF exposure due to the mobile phones usage. Finally, also modelling and simulations strategies were used for indoor exposure assessment, in order to estimate whole body and organ-related Specific Absorption Rate.

A recent study by Bhatt et al. [24] reviewed the available measurement tools for RF-EMF exposure assessment, highlighting main advantages and limitations of each solution. Moreover, a recent survey by Bolte [22] reviewed main sources of biases of uncertainties when using exposimeters for personal and micro-environments surveys, and assessed temporal and spatial variability and the reproducibility of measurements. These aspects were found to be fundamental in order to make any general conclusion on the exposure, providing that exposure prediction based on that results an accurate description of real life situations [22].

Last but not least, it is well known [25] that an important aspect of RF-EMF measurements is related to the handling strategy of non-detect, i.e., of the values below the lower detection threshold of the device used for the measure. This issue is often details in the considered papers (see, e.g., [26,27]). More details about the assessment strategies are reported in the following sections, with particular attention paid to the differences in the measurements and simulations protocols applied in different studies that could influence results and make difficult to compare outcomes of different studies.

#### 3.3.1. Spot and Long-Term Measurements

One of the most widely used techniques used to assess the exposure in indoor environments involves spot measurements of the EMF levels in selected locations by narrowband and broadband methods. Broadband measurements give the estimation of the whole field level due to all the sources in the observed radiofrequency band, whereas narrowband measurements highlight the contribution of each type of source to the overall exposure level. Spot measurements in indoor environments have been carried out in the range from several MHz to 10 GHz, with many studies focused on exposure in the frequency range of mobile telecommunications (base stations). 

Large-scale measurement campaigns based on spot and long-term RF monitoring systems have been carried out, but, even when the same tools are used, measurements protocol used in different studies were often completely different. First, the locations where to conduct measures were selected with different approaches depending on the study: some studies established some representative positions for each indoor environment [28], with fixed distances from the indoor sources or following a predefined measurement path [29], while other approaches based their investigation on a random sampling [19,30].

Second, as the level of exposure for a certain location is usually obtained averaging the values recorded during a time interval, the sampling time used by the instrument and the total temporal duration of the measurements in each locations represent other factors of variability among results of different studies [24]. The way in which the measurement of the exposure level took into account the time variations could lead to significant variabilities among the obtained values especially when considering sources emitting quasi-stochastic signals like the wireless local area networks WLAN [31]. Moreover, also the part of the day (morning/afternoon) and the day of the week (working days vs. weekend) play their role in exposure assessment, considering that they are both known to influence the exposure [5,6,24].

Third, a last source of variability among different studies were the exposure metrics (electric field amplitude vs. power density) and the descriptive statistics used to represent the results, such as mean, median, maximum or minimum values, or percentiles of the distribution. Moreover, some studies dealt with both indoor and outdoor environments, sometime presenting separately the results of the two different cases, but, in other cases, reporting them in a mixed form, making difficult the comparison among results from different studies.

#### 3.3.2. Personal Measurements

Data collected from spot measurements in indoor environments do not provide a good point of view about the exposure levels of individuals who are often exposed to multiple sources at the same time and, during their daily routine, move from one place to another. Data on the EMF exposure of individuals can only be obtained by using personal exposure meters (PEM), i.e., portable devices that are worn on the body to continuously measure for an interval of time the incident electric fields at the location of a subject wearing the device. These devices are calibrated in free space but are used on the body, therefore an uncertainty analysis is crucial, mainly based on: (1) body shielding, i.e., perturbation of real EMF exposure levels due to the presence of the human body in the immediate proximity; (2) residual uncertainties due to calibration; (3) how well the shaped frequency response of probes follows sensitivity variation with frequency; and (4) measurement artefacts. In addition, measurements with PEM tend to underestimate or overestimate the actual RF EMF exposure, depending on the frequency bands. A potential solution would be to determine correction factors based on the calibrations in order to modify the measurement results [21,32].

In indoor scenarios the shadow from the body, i.e., the perturbation of real EMF exposure levels due to the presence of the human body in the immediate proximity, is partly decreased by multi-pathing caused by reflections to walls and objects [33], thus the systematic measurement error depends on the size of the room: the smaller the room, the smaller the body shadow effect. Moreover, the response depends highly on the position of the exposimeter on the body, mainly on the separation distance between the exposimeter and the body and the tissue permittivity [34]. 

Studies using personal measurements focused on the identification of typical exposure levels in indoor environments (see, e.g., [20]) and on population surveys by questionnaires in which the personal exposure distribution in the population of interest was determined (see, e.g., [3]). Even if some studies focused on developing ad hoc standard study protocols for personal measurements campaigns [35], a wide variety of strategies for the recruitment of the study participants, either trained researcher or volunteers of different ages, as well as the data analysis methods differ between studies, making a direct comparison of their results extremely difficult.

#### 3.3.3. Most Common Used Measurement Devices

About the devices used in the considered studies, most spot measurement studies were conducted using spectrum analyzer, isotropic antennas and different narrowband devices, while most personal measurement studies were conducted using narrow band portable devices and personal distributed exposimeters. Commonly used exposimeters include various versions of EME Spy (SATIMO, Courtaboeuf, France), ESM 140 (Maschek Electronik, Bad Wörishofen, Germany), Narda exposimeters (Nardalert S3, RadMan, and RadMan XT) [Narda Safety Test Solutions, New York, NY, USA], ExpoM-RF (Fields at Work GmbH, Zürich, Switzerland), and personal distributed exposimeters (PDEs) (Ghent University/iMinds, Ghent, Belgium) [36]. The Narda exposimeters are broadband exposimeters, whereas the others are narrowband. All these devices have very different characteristics, in terms of number of frequency bands that can be discriminate (e.g., 20 bands in EME Spy 200, 16 bands for ExpoM-RF, 8 bands for the ESM 140), the total bandwith, the sampling interval (e.g., equal to 0.5–10 s for ESM 140 and Nardalert S3, 4–255 s for the EME Spy 200, and 3–6000 s for ExpoM-RF), sensitivity in terms of lower detection threshold, size and weight. For a detailed description of all these devices characteristics, see [24].

#### 3.3.4. Modelling and Simulations

A high number of studies investigated the RF-EMF exposure by modelling and simulations methods. The most accurate method to perform computational estimation is directly solving the Maxwell’s equations: the EMF induced in human tissues are directly calculated by using full wave techniques such as Finite Difference Time Domain, or equivalent methods. However, as the large requirements in terms of memory use and the high computational cost make these methods inappropriate for large area calculations, most of the previous studies based on computational methods focused on the analysis of cases in which the sources are placed close to the human body (see, e.g., [37]), and only few of them specifically addressed the specificity of the exposure when considering indoor environments. 

An alternative approach based on modeling and simulations to assess exposure in large spaces as indoor environments is represented by using methods traditionally used for indoor propagation prediction models, such as ray tracing and ray launching methods. These approaches offer a good approximation with a lower computational cost. Ray tracing and launching methods consist in launching rays from an antenna to a scenario or to a part of it, choosing radiation pattern; every ray will result in a reflected, transmitted or diffracted ray, depending on the stage element to impact and how the impact occurs. The rays on the impact surface will contribute to the total received field at that point, depending on the electromagnetic parameters of the materials interacting with the propagating waves. Implementation based on a combination of geometric optics and physical optic allowed using ray tracing in order to characterize wireless propagation and consequently RF-EMF exposure in very complex indoor scenarios [38,39].

A further method combining heuristic predictions and ray tracing methods, based on the calculation of the dominant path between transmitter and receiver, was used in a large number of studies dealing prediction of downlink exposure, i.e., exposure due to the power received by a user device, and uplink exposure i.e., exposure due to the power emitted by a user device, for indoor wireless networks planning, thus giving an indirect estimation of the RF-EMF exposure in different indoor environments, such as offices [40] and trains [41]. 

A step ahead in the assessment of the exposure in indoor environments considering realistic and uncertain scenarios is represented by stochastic dosimetry. Stochastic dosimetry is an innovative approach based on statistics that, starting from a set of observations obtained either by deterministic methods such as FDTD or ray tracing or by measurements, builds surrogate models able to replace the heavy computational problem with analytical problems. This approach was recently used to assess the exposure to RF-EMF sources in indoor environments considering highly uncertain scenarios [42].

### 3.4. RF-EMF Exposure Levels

This section summarize the levels of exposure reported by the different studies, divided by different types of indoor environments. For sake of homogeneity, all the exposure levels were reported also as a percentage of the reference level according to the International Commission on Non-Ionizing Radiation Protection (ICNIRP) guidelines [43] for the corresponding frequency band. When considering levels of exposure due to the wide RF-EMF frequency band, the values were compared to the lowest (i.e., the strickest) ICNIRP reference level of the whole band, in order to account for the worst case.

#### 3.4.1. Exposure in Public Places

##### Working Places

As people spend a large amount of their time in the office during working hours, many previous studies focused on the assessment of the exposure in office buildings, with particular attention paid to the presence of wireless local area networks (WLANs) [17]. As an example, Ibrani et al. [44] used personal exposure measurements for assessing the exposure of people working in the offices of some companies which are characterized by extensive use of wireless technologies. The measurements were recorded during working hours, after working hours and during the weekends using personal exposimeter that measured the total level of exposure between 88 and 5850 MHz, and the relative contribution of 14 frequency bands used for wireless communications. The results showed that the total mean power density value of the working day was 0.524 mW/m^2^ (0.4 V/m in terms of electric field, corresponding to 1.5% of lowest ICNIRP reference level for the 88–5850 MHz frequency band). During the weekend, for the same exposure hours as for the working day, the mean power density value was 0.828 mW/m^2^ (0.56 V/m, 2% of lowest ICNIRP reference level for the 88–5850 MHz frequency band, mainly due to the exposure to the GSM (DL) system (0.500 mW/m^2^, 0.43 V/m in terms of electric field, 0.7% of the ICNIRP reference level). The study included the exposure due to Wi-Fi in the frequency band between 5.1 and 5.8 GHz, finding that its contribution was relatively small (0.046 mW/m^2^, 0.13 V/m in terms of electric field, 0.22% of the ICNIRP reference level), and confirming that the main contributors to RF-EMF exposure are mobile phone systems (0.255 mW/m^2^, 0.31 V/m in terms of electric field, 0.53% of the ICNIRP reference level) and DECT systems (0.178 mW/m^2^, 0.26 V/m in terms of electric field, 0.45% of the ICNIRP reference level). The authors concluded that the total amount of personal exposure, surprisingly higher during the weekend that during the working days was mainly dependent on the personal habits of the use of such systems, rather than indoor workplace specifics.

Schmid et al. [45] investigated RF-EMF exposure in offices due to indoor used wireless communication technologies such as WLAN (2400–2483 MHz), Bluetooth (2400–2483 MHz) and Digital Enhanced Cordless Telecommunications (DECT) systems (1880–1900 MHz). Results showed that under usual conditions, the resulting spatially (over body dimensions) averaged and 6-min time-averaged exposure was below 0.1% of the reference level for power density according to the International Commission on Non-Ionizing Radiation Protection (ICNIRP) guidelines [43], and that none of the devices considered in this study exceeded the limits according to the ICNIRP guidelines. 

Gryz et al. [34] investigated RF-EMF exposure in offices (where exposure can be caused by various transmitters of local fixed indoor and outdoor wireless communication systems), and in buildings (in urban and rural areas in various regions of Poland), using personal exposure assessment. The main sources of exposure were found to be mobile phone base stations (BTS) and radio-television transmitters (RTV). The frequency composition in a particular office depends on the building’s location. The E-field recorded during 24 h in buildings from the outdoor BTS had median and 75th percentile values lower than 0.19 and 0.25 V/m (0.5% and 0.6% of the corresponding ICNIRP reference level), in urban areas and lower that 0.05 and 0.09 V/m (0.1% and 0.2% of the corresponding ICNIRP reference level) in rural areas. In buildings equipped with indoor BTS antennas the E-field median and 75th percentile values were lower than 1 V/m, and 1.8 V/m (2.6% and 4.6% of the corresponding ICNIRP reference level), respectively. Whereas in urban and rural areas, the median and 75th percentile values of the E-field recorded in buildings located near the RTV (within 1 km) where lower than 1.5 and 3.8 V/m (5% and 14% of the corresponding ICNIRP reference level) and 0.4 and 0.8 V/m (1.4% and 2.8% of the corresponding ICNIRP reference level), for radio FM band or for TV bands, respectively.

A survey from Lunca et colleagues [46] on peak RF emissions in typical office environments found a peak power density equal to 54 mW/m^2^ (4.5 V/m in terms of electric field, 7% of the ICNIRP reference level) at a distance of 1 m from a Wi-Fi access point operating at 2.45 GHz, which would correspond to an Effective Isotropic Radiated Power (EIRP) from the access point of about 0.6 W.

In a study by Amizadeh et al. [36] indoor WiFi-5G was measured in an office building in Ghent, Belgium using personal exposimeters (PEMs). Results showed maximum and mean power density values equal to 8.9 mW/m^2^ and 165.8 μW/m^2^ (1.8 V/m and 0.25 V/m, in terms of electric field strength).

Three studies [13,14,16] focused on the assessment and comparison of the RF-EMF exposure of a mobile phone user that is either connected to an femtocell or a conventional macrocell base station while in an office environment. Results of the three studies showed that, in average macrocell coverage and mobile phone use-time conditions, the use of the femtocell improved reception quality indoor [13] while the total exposure was reduced by using a femtocell, mostly due to the significant decrease in the output power of the mobile phone [14,16].

Finally, a study by Chen and colleagues [47] focused on the RF-EMF exposure due to a femtocell in a typical office by simulation, without considering the exposure due to external sources such as base station for telecommunications of indoor sources different from the femtocell, such as mobile phones. In this study the finite-difference time-domain (FDTD) method was used to calculate electric fields emitted from a long-term evolution (LTE) femtocell, with and without the presence of 20 people and furniture. Simulated electric fields at most of the locations on the horizontal plane with a height of 1.0 m above the floor are found in the range of −10 to −30 dB V/m, which means a good signal will be picked up in the office. The maximum power density emitted from the LTE inside the office and maximum localized SAR induced in a standing person were found to be far below the IEEE safety standard for public exposure in uncontrolled environments [48].

##### Educational Buildings

Many studies focused on the RF-EMF exposure levels in buildings hosting educational activities, from kindergartens and nursery to universities. As an example, an extensive study of RF exposures from Wi-Fi access points and laptops used in schools in the UK was reported by Peyman and colleagues [49,50]. In [49], the authors investigated the electric field strengths from 15 different laptops and 12 different access points, typical of those used in UK schools, during Wi-Fi transmission. The maximum electric field strength values recorded at 0.5 m around laptops and access points were 2.9 mV/m and 5.7 mV/m, respectively, indicating that the field strengths from access points were generally higher (almost double) compared to those from laptops. The results also demonstrated that the electric field strength reduced rapidly with distance for all Wi-Fi devices. The corresponding maximum power density values for the laptops and access points at 0.5 m were 22 mW/m^2^ and 87 mW/m^2^, respectively, decreasing to 4 mW/m^2^ and 18 mW/m^2^ at 1 m distance. In the second part of the study [50] the authors examined Wi-Fi devices in a small sample of schools in the UK, with measurements being performed in both primary and secondary schools during lessons in which a range of tasks were being performed. The tasks included internet based interactive learning, emails, web browsing, and downloads. In all the networks examined, the duty factors of individual laptops, ranging from 0.02 to 0.96%, were considerably lower than those of the access points, with a range from 0.06 to 11.67%. Applying these duty factors to the earlier laboratory measurements [49] resulted in maximal time-averaged exposure at 0.5 m distance of 220 μW/m^2^ (22 mW/m^2^ with 1% duty factor) from individual laptops and 22 mW/m^2^ (87 mW/m^2^ with 12% duty factor) from access points. Even in the unlikely event that all 30 laptops in a classroom transmitted bursts simultaneously at the maximum 0.5 m distance power density, with the maximal duty factor of 1% measured in the present study, time-averaged exposure values from all laptops would only be 6.6 mW/m^2^. This exposure, added to the maximum time-averaged exposure from an access point at 0.5 m would only give 16.6 mW/m^2^. It has to be noticed that in these works [49,50] the assessment was focused on the RF-EMF emitted by Wi-Fi access points and laptops, discarding all other sources, placed indoor or outdoor, that may be influence the levels of RF-exposure in schools.

The study by Hedendahl et al. [18] focused on the assessment of the levels of RF-EMF exposure in schools due to the use of Wi-Fi systems, considering also the contribution due to mobile phones and external base stations. The assessment was carried out by measuring the teachers’ RF exposure during their activity at school in order to approximate the children’s exposure. Every teacher measured from 6 to 31 h, resulting in a total of 255 h of observations. The mean exposure to RF radiation ranged from 1.1 to 66.1 μW/m^2^ (0.02 V/m and 0.16 V/m, in terms of electric field, corresponding to 0.07% and 0.6% of the ICNIRP reference level), with higher levels during lessons when laptops or mobile phones were actively used. The maximum values were found to be higher when connecting to mobile phone base stations outside of the school building, up to 82.8 μW/m^2^ (0.18 V/m, in terms of electric field, corresponding to 0.45% of the ICNIRP reference level) from uplink from GSM 1800, 3G or 4G. The maximum level of exposure due to the downlink from the same base stations was 3.3 mW/m^2^ (0.04 V/m, in terms of electric field, corresponding to 0.09% of the ICNIRP reference level). Maximum peaks when considering only the Wi-Fi were equal to 4.5 μW/m^2^ (0.04 V/m, in terms of electric field, corresponding to 0.07% of the ICNIRP reference level) and 3.3 μW/m^2^ (0.035 V/m, in terms of electric field, corresponding to 0.06% of the ICNIRP reference level), for 2.4 GHz and 5 GHz, respectively. Total means values considering all teachers were equal to 2.8 μW/m^2^ (0.03 V/m, in terms of electric field, corresponding to 0.05% of the ICNIRP reference level) for Wi-Fi 2.4 GHz and to 3.1 μW/m^2^ (0.034 V/m, in terms of electric field, corresponding to 0.06% of the ICNIRP reference level) for 5 GHz. 

A study by Karipidis [51] focused on the assessment of the RF levels from Wi-Fi and other sources in 23 schools in Australia. All measurements were performed via appointment mainly during school hours between 8.30 a.m. and 3.30 p.m.; one school was measured during school holidays and another during the school’s sports day where all the students were off campus. Results showed that the mean and peak RF levels when considering just the Wi-Fi contribution in locations occupied by children in the classroom were of the order of 10^−4^ and 10^−2^% of the ICNIRP exposure guidelines [43]. The authors concluded that the typical RF exposure of children from Wi-Fi at school was lower than the exposure due to other sources in the environment.

In a study by Bhatt et al. [52] the environmental and personal RF-EMF exposure was assessed in kindergarten considering the contribution of 16 frequency bands from 88 MHz to 5.8 GHz. The environmental exposure was assessed by spot measurements in five different points in each classroom, each one lasting 6 min. The personal measurements involved 10 children from five different kindergartens and were performed between 08.30 to 13.30 h (October–December 2015). The median value of the electric field obtained by environmental measures in the indoor facilities of the kindergartens was equal to 127 mV/m considering all the sources (i.e., all the contribution from the 16 considered frequency bands). As to the mobile phone base station exposure, the authors found levels equal to 66 mV/m for the downlink exposure (0.17% of the lowest ICNIRP reference level for the downlink band) and equal to 25 mV/m for the uplink exposure (0.06% of the lowest ICNIRP reference level for the uplink band). The Wi-Fi 2.4 GHz caused a level of exposure equal to 20 mV/m (0.03% of the of the ICNIRP reference level). Similarly, for personal exposure, median values were found equal to 81 mV/m (all bands, 0.29% of the lowest ICNIRP reference level for the whole frequency band), 62 mV/m (total mobile phone base station downlinks, 0.16% of the lowest ICNIRP reference level for downlink frequency band), 21 mV/m (total mobile phone base station uplinks, 0.05% of the lowest ICNIRP reference level for downlink frequency band), and 9 mV/m (Wi-Fi 2.4 GHz, 0.02% of the ICNIRP reference level). The measurements showed that environmental RF-EMF exposure levels exceeded the personal RF-EMF exposure levels at kindergartens. Moreover, the authors found that the median exposure levels for those kindergartens <300 m from the nearest mobile phone base station were significantly higher compared with the medians for those >300 m away.

In a recent study by Kurnaz et al. [53] extensive short-term/band-selective and long-term RF-EMF measurements were conducted at 92 primary and secondary schools in Turkey, in May, June, October and December, 2016. The short-term/band-selective measurements lasted 6 min in each point, while, after completing broadband and bandselective measurements, 24 h of E measurements were taken at the school with the highest average electric field strength (E_avg_). Results showed that the maximum E_avg_ was recorded, 2.34 V/m (8% of the lowest ICNIRP reference level for the considered frequency band), in October, the month in which more students were at school. The authors concluded that the measured E_avg_ levels recorded at 92 schools were below the ICNIRP guidelines [43]. The five main electric field strength (E) sources that had the most contribution in total *E* were LTE800, GSM900, GSM1800, UMTS2100 and WLAN services. It was also concluded from the long-term broadband measurement result that the number of active users affected the total E, and that the measured E levels were significantly higher in daytime than those of recorded in night-time.

A study by van Wel et al. [54] measured indoor RF-EMF levels to which children are exposed in a large number of primary schools in Amsterdam. Measurements in each classroom consisted of seven spot measurements of two minutes each, with sampling of 4 s, carried out after lessons, in the afternoon. Results showed average power density of 70.5 mW/m^2^, (0.16 V/m). Main contributors to total RF-EMF levels were mobile phone downlink and DECT signals. Over half of detected signals (56.3%) originated from outdoor sources (e.g., mobile phone downlink, broadcast). When looking at signals that originated from indoor sources (e.g., DECT, WiFi, mobile uplink), DECT was the main contributor, followed by mobile phone uplink. Most variance between measures was explained by differences between rooms.

Many other public places, such as small and large shops, shopping malls, railway stations and airports, have been investigated. However, most of the studies presenting results about the levels of exposure to RF-EMF in these facilities were not focused on a deep investigation about each single type of indoor environment, but included measurements in a high number of different places (results of these studies will be describe the “Comparison between different types of indoor environments” section). The only paper focusing on a large public indoor environment such a railway station was the survey by Hardell et al. [29], focused on the assessment of the levels of exposure in the Stockholm Central Railway Station. The authors found that total power density mean values for a walking round varied between 2.817 to 4.891 mW/m^2^ (1 V/m and 1.36 V/m in terms of electric field, 3.6% and 4.9% of the lowest ICNIRP reference level for the considered frequency band). GSM and UMTS 900 and 2100 downlink contributed to almost half of total exposure. Other major sources were LTE 800 downlink, GSM 1800 downlink and LTE 2600 downlink, while other sources were comparatively low.

#### 3.4.2. Exposure in Private Places

A high number of studies focused on the assessment of the exposure in residential environments, both using measurements and simulations tools. A residential RF exposure assessment was performed by Breckenkamp et al. [28] by measuring the electric field in fixed positions in bedrooms in 1348 households in Germany in 12 frequency bands from 88 MHz to 2.50 GHz, with the aim of investigating the contribution of different RF-EMF sources (FM radio, analogue TV and DVB-T, TETRA, GSM900 downlink, GSM1800 downlink, UMTS downlink, DECT, and wireless LAN and Bluetooth) to the total exposure. Measurements were carried out from 8 a.m. to 9 p.m. in 4 positions in each bedroom, and at each position 75 measurements were taken within 5 min. The first 5 of 75 measures in every position were deleted (resulting in 280 measuring values for every frequency range per household), Results showed that the exposure levels were often below the detection limit (electrical field strength: 0.05 V/m) of the dosimeter, while the total exposure varied, depending on residential characteristics (urban vs. rural areas) and on the floor of the building where measurements took place. DECT and WiFi accounted for 82% of total exposure level, with mean values of power density equal to 22.45 μW/m^2^ and 6.37 μW/m^2^ (0.09 and 0.04 V/m, in terms of electric field strength, 0.16% and 0.08% of the respective ICNIRP reference levels). Exposure from mobile phone base stations accounted for only 6.3% to total exposure, while the contribution of the uplink sources, such as mobile phones, was not included in the study.

Another cross-sectional study by Hutter et al. [55] in houses placed near to base stations in Austria showed that total RF EMF exposure measured in bedrooms of 336 households, including mobile telecommunication signals, was far below recommended levels, with maximum values of power density equal to 4.1 mW/m^2^ (1.24 V/m in terms of electric field strength); the greater portion of that exposure was from mobile telecommunications (geometric mean 73%). Overall 5% of the estimated maximum exposure levels were above 1 mW/m^2^. Distance of the houses from antennas was 24–600 m in the rural area and 20–250 m in the urban area. The mean value of electric field strength was slightly higher in a rural area (0.13 V/m) than in an urban area (0.08 V/m). This discrepancy was due to the fact that only those households were selected that were close to mobile phone base stations, and base stations in rural areas typically transmit higher power as they are required to transmit over larger distances.

A study by Burgi et al. [56] proposed a geospatial model to predict the radiofrequency electromagnetic field in indoor environments from fixed site transmitters for use in epidemiological exposure assessment, used in some of the studies described in the followings (see, e.g., [3]). Findings showed that mean value of the RF-EMF exposure in residential buildings was equal to 0.13 V/m.

In the study by Pachon-Garcia [57], the exposure due to Wi-Fi networks was analyzed in different locations of a two floor house located in a residential area, by moving the router-terminal devices in different positions, and considering different types of traffic which sent through the network. The results showed maximum background exposure to WLAN equal to 39 mV/m when the Wifi network under exam is turned off, and a maximum exposure equal to 2.6 V/m in the far field region of the transmitters when the WiFi network is turned on. Concerning the type of traffic, oscillations up to 10 dB were detected for exactly the same position, depending on the type of traffic was being sent. Differences around 62 dB in mean values between the different rooms of the house were found. All values were below the threshold of 61 V/m prescribed at 2100 MHz by the ICNIRP guidelines for general public [43].

In a study by Kottou and colleagues [58] EMF exposure level measurements in Greece were performed in a variety of indoor dwellings, in three different parts of the country. A total number of 4540 measurements were taken in a wide frequency range (50 Hz–2100 MHz) in three regions, i.e., Attica, Lesvos and Zakynthos. The duration of each measurement was set between 5 and 15 min. All measurements are presented as time averaged values. Results showed that maximum levels of exposure were, in most cases, below 0.5 V/m, with peak values above 1 V/m and up to 5.6 V/m occasionally observed.

A study by Hardell [59] assessed the exposure to RF-EMF in an apartment with a central location in Stockholm, located on the 6th floor, with a tower including a bedroom on the first floor of the tower (7th floor) and a conference room on the second and highest floor (8th) of the tower, at the same level as the roof of the building. Results were obtained by repeated measures in different locations in the apartment, during day and night, for total of indoor 71 h of measurements. Mean and median values of the exposure were found to be equal 1.766 mW/m^2^ and 1.051 mW/m^2^ (0.81 V/m and 0.6 V/m).

In a follow up study by Tomitsch et al. [19], the levels of exposure due RF-EMFs in different frequency bands (discarding the mobile phone uplink signals) were measured in 219 bedrooms in Lower Austria in 2006, 2009 and 2012. The authors analyzed both the differences among measurements obtained in different years, and the influence of different urbanization levels of the area in which the residential buildings were placed on the levels of exposure. The total exposure was due for a 15% to indoor sources and for the 85% to outdoor sources. Results showed that the arithmetic mean of the RF-EMF electric field decreased from 0.51 V/m in 2006, to 0.46 V/m in 2009, and to 0.37 V/m in 2012 (respectively, 1.76%, 1.59% and 1.28% of the lowest ICNIRP reference level for the considered frequency band). On the contrary, the median values were found to increase from 0.10 V/m in in 2006 to 0.14 V/m in 2012 (respectively, 0.34%, and 0.48% of the lowest ICNIRP reference level for the considered frequency band). This difference between the arithmetic mean and median was because the arithmetic mean also considers high outliers whereas the median represents the main part of the measurement spots. The highest increases from 2006 in the contribution to the RF-EMF exposure were found for UMTS (from 0 to 0.52 μW/m^2^, equal to 0.01 V/m in terms of electric field strength, 0.02% of the ICNIRP reference level) and wireless local area networks (from 0 to 0.46 μW/m^2^, equal to 0.01 V/m in terms of electric field strength, 0.02% of the ICNIRP reference level), while a decrease was observed in the exposure level in the frequency range of digital enhanced cordless telecommunications telephones (from 1.80 to 0.19 μW/m^2^, respectively equal to 0.3 and 0.01 V/m in terms of electric field strength, 0.07% and 0.02% of the ICNIRP reference level). Comparing the levels of exposure in the household placed in rural areas, small towns, suburban and urban areas, the results showed higher total RF-EMFs in urban (117.73 μW/m^2^, equal to 0.21 V/m in terms of electric field strength, 0.75% of the lowest ICNIRP reference level for the whole frequency band considered) than in rural (median 34.52 μW/m^2^, equal to 0.11 V/m in terms of electric field strength, 0.4% of the lowest ICNIRP reference level for the whole frequency band considered) areas. For total mobile phone downlink signals the median was 5.22 μW/m^2^ (equal to 0.04 V/m in terms of electric field strength, 0.11% of the ICNIRP reference level) in rural areas, 39.18 μW/m^2^ (equal to 0.12 V/m in terms of electric field strength, 0.05% of the ICNIRP reference level) in small towns, 33.49 μW/m^2^ (equal to 0.11 V/m in terms of electric field strength, 0.04% of the ICNIRP reference level) in suburban areas and 51.37 μW/m^2^ (equal to 0.14 V/m in terms of electric field strength, 0.05% of the ICNIRP reference level) in urban areas. The biggest differences between rural and urban areas occurred in UMTS (factor 77.5), GSM1800 (factor 32.5) and WLAN (factor 10.9). Considering the signal dominance, GSM900 was dominant in all four areas. Finally, comparing the results obtained at different floors, results showed that the highest floors are more often in line of sight to mobile phone base stations and in the main lobe of the antenna resulting in higher power flux densities than those measured at the lowest floors.

Another study by Viel and colleagues [60] was aimed at characterizing the distribution of residential exposure due to antennas, to assess how exposure to RF fields varies with distance from these point sources, and to test the association of RF exposure with the level of urbanization of the area by using personal exposure meters. A total of 200 randomly selected people were enrolled. Each participant was supplied with a personal exposure meter for 24 h measurements, and kept a time–location–activity diary. Two exposure metrics for each radiofrequency were then calculated: the proportion of measurements above the detection limit (0.05 V/m), and the maximum electric field strength. Residential address was geocoded, and distance from each antenna was calculated. Results showed that the recorded field strength was often below the detection level (0.05 V/m). The maximum electric field strength was always lower than 1.5 V/m (i.e., lowest of the 5% of the ICNIRP reference level). Exposure to GSM and DCS peaked around 280 m and 1000 m from the antennas, while a downward trend was found within a 10 km range for FM. Conversely, the exposure due to UMTS and TV signals did not vary with distance.

Two recent studies [12,42] assessed the exposure of a child to either a WLAN and a femtocell placed in a representative room, when both the child and the source are placed in an uncertain position. In both studies, a stochastic dosimetry approach based on low rank tensor approximation was used to develop surrogate models for fast estimating the Specific Absorption Rate (SAR) in all the possible positions of the sources and the child. Results showed that, for all the possible positions of the sources and the child in the room, the exposure values were significantly below the levels indicated in the guidelines of the International Commission of Non-Ionizing Radiation Protection (ICNIRP) guidelines for general public exposure [43], with probability of reaching SAR values higher than 1% of the ICNIRP guidelines value was lower than 0.006.

#### 3.4.3. Exposure in Public and Private Transportation

A high number of studies focused on the assessment of the RF-EMF exposure on transportation could be found in literature, due to the peculiar conditions of being densely populated environments where wireless connection quality could be not stable in time. 

In a study by Sagar et al. [61] the RF-EMF exposure was quantified using portable devices with a high sampling rate in different public transport vehicles, in Switzerland, Ethiopia, Nepal, South Africa, Australia and the United States of America. The measurements were taken for about 30 min with a sampling rate equal to 4–5 s, depending on the instrument. Across various modes of public transport, the authors found that total exposure, arising from uplink and downlink transmission, broadcasting and WiFi, ranged between 0.37 V/m (1.32% of the lowest ICNIRP reference level for the considered frequency band) in bus in Switzerland and 0.86 V/m (3% of the lowest ICNIRP reference level for the considered frequency band) in auto rickshaw in Nepal. Considering separately the different contribution to the exposure, the authors found that uplink transmission was the most relevant exposure source in trains, with the highest values in Switzerland (with a maximum value of 0.47 V/m, 1.24% of the ICNIRP reference level, in Zurich), while, for the other types of vehicles the most relevant source of exposure was found to be the downlink one, with values ranging from 0.28 V/m (0.74% of the ICNIRP reference level) on the bus in Switzerland to 0.85 V/m (2.25% of the ICNIRP reference level) on the auto rickshaw in Nepal. Relevant differences were observed between the uplink exposure across public transportation in Switzerland and Nepal probably because Switzerland is technologically more advanced than Nepal where fewer people traveling on public transportation use smartphones.

In a similar study by Thielens et al. [62], the authors investigated the repeatability and representativeness of personal RF-EMF exposure measurements, across different micro-environments, including different public transport vehicles. The authors observed large variations in RF-EMF exposure depending on the type of transportation that is used, with the lowest total levels of exposure in cars (E_rms_ equal to 0.18 V/m, equal to 0.64% of the lowest ICNIRP reference level for the considered frequency band) when not using any RF-EMF emitting devices, and the highest total exposure in tram and trains (E_rms_ equal to 0.38 V/m and 0.23 V/m, respectively, equal to 1.36% and 0.82% of the lowest ICNIRP reference level for the considered frequency band). 

A study by Gryz et al. [63] investigated the levels of RF-EMF exposure inside the metro infrastructure in Warsaw by frequency-selective exposimeters. Results showed that most of the exposure was due to mobile phones base transceiver stations and handsets used by the passengers (GSM 900 and UMTS 2100) and to local wireless Internet access (WiFi 2G). The highest values were observed for GSM900, with median and 75th percentile values of the E-field equal to 0.22 V/m and 0.37 V/m for the downlink (equal to about 0.5% and 0.9% of the ICNIRP reference level) and to 0.28 V/m and 0.47 V/m for uplink of the handsets (equal to about 0.7% and 1.1% of the ICNIRP reference level). Broadband measurements, including the dominant signal emitted by staff radiophones (151 MHz), showed that the level of this exposure of engine-drivers does not exceed 2.5 V/m.

Two recent studies [64,65] assessed the impact on the passengers’ exposure of the deployment of a miniature small cell or a femtocell in a train car in order to improve the coverage and the capacity of a mobile network service on the train. Three sources of RF-EMF were considered: (1) the subject’s mobile phone (a near-field source); (2) the base station to which the mobile phone was connected (a far-field source), and in case of the small-cell scenario; (3) the macrocell as well, as it is still present and radiating. The highest exposure values in terms of median Specific Absorption Rate (SAR) equal to 61 μW/kg were found for the GSM 900 technology, without the presence of small cell/femtocell on the train. In both studies the authors found that by connecting to a small cell of to a femtocell, the exposure of the user could realistically be reduced. In another study by the same authors [41] the contribution of RF-EMF originating from other people’s devices to total own whole-body absorption in a train environment has been assessed in a simulation study. Absorption in a train environment due to base station’s downlink is compared with absorption due to uplink of the user’s own mobile device and absorption due to the uplink of 0, 1, 5, or 15 other nearby active users. Results showed that the contribution of other in-train users is not negligible: 15 other users connected to a GSM900 macro cell base station can induce absorption rates up to 14.3 μW/kg, i.e., 24% of the absorption rate induced by a user’s own device, equal to 60.6 μW/kg. This corresponds for the considered scenario to a contribution of 19% to the total absorption rate (equal to 74.9 μW/kg) when calling yourself and a contribution of 100% when not calling yourself. A UMTS femtocell deployment in this environment drastically reduces total absorption and makes the other users’ contributions to total absorption negligible.

Some studies focused on the assessment of the RF-EMF exposure inside road vehicles by using computational methods [66,67,68]. In the study by Harris and colleagues [66] numerical dosimetry for adult and children models exposed to several wireless communication systems (UMTS, WiMax, and Bluetooth) localized in different positions within a partly shielded environment represented by a realistic car model, considering different exposure scenarios. Results showed that for a single operating device, the maximum whole body SAR for adults and for children exposure is 1.39 and 1.58 mW/kg, respectively. For two simultaneously emitting sources, these values increase to 2.04 and 2.13 mW/kg. For all considered exposure scenarios, the whole-body average SAR is more than 40 times lower than the ICNIRP general public exposure limit [43]. The maximum 10 g-averaged localized SAR was found in the head, trunk, and limbs, with maximum SAR values in the limbs, at least 10 times lower than ICNIRP limits [43]. for both adult and children models. The whole-body average SAR values for children are found to be typically 1.1–1.3 times higher than those of adults. Under several exposure scenarios, the local SAR in the limbs of children models is 2–3 times higher than corresponding values in adult models. Analyzing the power density distributions within the car for one, two, and three simultaneously emitting devices, the authors found that the homogeneity of the power density distribution increases with increasing number of simultaneously operating transmitters, thus suggesting that the use of several wireless communication devices within a car leads to exposure levels that are several orders of magnitude below the ICNIRP general public exposure limit [43].

In the paper by Leung et al. [67] the specific absorption rate (SAR) induced in mobile phone users inside a vehicle was evaluated by finite-difference time-domain method in different scenarios, including handedness, passenger counts, and seating locations. The authors simulated the situation in which only one of the passengers is talking on a mobile phone (i.e., the user) at a fixed location for all the studied cases, while the rest of the passengers were without any wireless communication device. The output radiation power of the mobile phone was set to 2W for modeling the worst-case scenario in mobile communications. The whole body SAR values in mobile phone users in free space were compared to those inside a vehicle; results illustrated that the maximum SAR induced for mobile phone users in a vehicle is 5% higher than those in free space, with no significant difference for the handedness. The findings demonstrated that the change of passenger count inside the vehicle can alter the maximum SAR value induced in the bodies of passengers. In addition, when a passenger sits next to a mobile phone user, the maximum SAR value of that passenger can be up to about 5% of that of the mobile phone user, with maximum observed value (not averaged) equal to 0.233 W/kg.

Two recent studies based on 3D ray-launching method, estimated of electric field amplitude in a car [69] and in a commercial passenger airplane [39]. As to the car, the exposure to ZigBee, GSM and UMTS sources has been estimated both by simulations and by measurements. Higher electric field levels have been observed using ZigBee compared with UMTS or GSM, due to the fact that ZigBee emitting power configuration has been set to 63 mW value, whereas mobile terminals due to power control mechanisms emit at minimum power levels. In all cases, the systems comply with reference levels found in the recommendations proposed by ICNIRP [43]. The obtained values were strongly influenced by topology, morphology and materials of the confined vehicular scenario, which can give rise to high signal levels and hence, specific regions or hot spots to monitor.

As to the exposure in airplane [39], the authors used the 3D ray-launching method to assess the exposure to a radio frequency source operating at 900 MHz, 2.4 GHz and 5 GHz in the main cabin. The topology as well as the morphology of the main cabin of the airplane were known to play a key role in the obtained estimation values, since multipath components are the dominant contribution to the overall mechanism of radio propagation losses within the scenario. Moreover, the strong metallic content within the main cabin emphasized the multipath components, leading to strong signal variation, especially in the vicinity of the radiation sources. Estimations of the exposure in different exposure scenarios (with passenger seats and without passenger seats), showed that there were some regions of the cabin in which compliance with ICNIRP [43] as well as IEEE C95.1 standards [48] was not fulfilled, as values higher than 80 V/m were observed for 5 GHz and higher than 160 V/m for 2.4 GHz, for height from the ground equal to 1.7 m.

#### 3.4.4. Comparison between Different Types of Indoor Environments

A high number of studies did not focus on a single type of indoor environment, but investigated the exposure in a high number of different public and private places. These studies usually involves large number of measurements, both spot and personal, involving adults and children and considering different aspects of the exposure, such the temporal variability of the differences among different countries. Only in some cases the RF-EMF exposure values for specific environment were reported in details. 

In the study by Vermeeren et al. [70] the authors assessed and compared spatial and temporal RF exposure in different indoor microenvironments (schools, nurseries, offices and homes) in Belgium and Greece. At every location, broadband and narrowband measurements were performed in two rooms. The broadband and narrowband measurements were performed during daytime on weekdays, while the temporal measurements lasted for a whole week. While mobile telecommunications were most present in all indoor microenvironments together with radio broadcasting (FM), the highest average exposures were found in office environments (1.1 V/m in Belgium and 0.7 V/m in Greece, equal to 4% and 2.6% of the ICNIRP reference level) and lowest in homes (0.3 V/m in Belgium and 0.4 V/m in Greece equal to 1.1% and 1.4% of the ICNIRP reference level), mainly due to mobile telecommunications. The highest electric-field values were obtained in urban environments and lowest in rural environments. Concerning the temporal measurements, the time-evolution of the total signal over 24 h was mainly due to variations of mobile telecommunication signals in all indoor microenvironments. In offices, the time evolution of the total signal was influenced also by TV signals.

Juhász et al. [71] used personal exposimeters for 24 h to assess the levels of exposure in elementary schools, kindergartens, day nurseries, and, as control cases, in offices. As a first finding, most of the recorded data were below the detection limit of the measurement tool, equal to 0.05 V/m. The authors reported that children exposures were comparable to the worktime exposure of adults. Electric-field values due to WiFi 2G were found to be higher in kindergartens than in schools.

Markakis and Samaras [72] assessed the exposure levels in different microenvironments (offices, bedrooms, living rooms, schools) in Greece by personal dosimeters. The measurements took three days in 40 different locations selected, both in the urban and suburban area of Thessaloniki. Mean exposure levels in the mobile downlink frequency bands were found to be equal to 0.256 V/m, 0.259 V/m and 0.131 V/m, for GSM, DCS and UMTS, respectively, while maximum values were found equal to 0.38 V/m, 0.3 V/m and 0.28 V/m, for GSM, DCS and UMTS, respectively. Results showed that signals from mobile base stations were dominant in workplaces and schools, whereas wireless phones and computer networks were most influential in home environments. 

In [73] the exposure to RF-EMF was assessed in schools, homes, public places located in urban environments and offices by performing spatial broadband and narrowband measurements, providing 6-min averaged electric-field strengths. 94% of the broadband measurements were below 1 V/m (3.5% of the lowest ICNIRP reference level for the considered frequency band), while the highest value was measured in offices and was 3.5 V/m (12.5% of the lowest ICNIRP reference level for the considered frequency band). Lowest fields were measured in homes and the highest percentage of measurements below 0.20 V/m (sensitivity of probe) occurred in homes, namely 67%. For the schools, 92% of the measurements were below 1 V/m and 56% were lower than the sensitivity level of the broadband probe (0.2 V/m). The average and maximal total electric-field values in schools, homes, generic public places and offices, were equal to 0.2 and 3.2 V/m in schools (0.7% and 11.4% of the lowest ICNIRP reference level for the considered frequency band), 0.1 and 1.1 V/m in homes (0.36% and 3.9% of the lowest ICNIRP reference level for the considered frequency band), and 0.6 and 2.4 V/m in public places (2.1% and 8.6% of the lowest ICNIRP reference level for the considered frequency band), and 0.9 and 3.3 V/m in offices (3.2% and 11.8% of the lowest ICNIRP reference level for the considered frequency band). In the considered schools, the highest maximal and average field values were due to internal RF-EMF sources (WiFi), while in the considered homes, public places, and offices, the highest maximal and average field values originated from telecommunication signals. The lowest exposure levels were observed in homes. Internal sources, such as the WiFi, contributed on average more indoors (31.2%) than outdoors (2.3%), while the average contributions of external sources (broadcast and telecommunication sources) were higher outdoors (97.7%) than at indoor positions (68.8%). In indoor measurements, FM, GSM, and WiFi dominate the total exposure. The average contribution of the emerging technology LTE was only 0.6%.

In a study by Beekhuizen et al. [74], 15-min spot measurements in 263 rooms in 101 primary schools and 30 private homes in Amsterdam, the Netherlands were assessed. The authors found an overall power density with mean value equal to 0.041 mW/m^2^ (0.12 V/m in terms of electric field strength, equal to 0.44% of the ICNIRP reference level), with the GSM900, GSM 1800 and UMTS contributing to exposure levels equal to 0.02, 0.012 and 0.01 mW/m^2^, (0.09, 0.07 and 0.06 V/m in terms of electric field strength, equal to 0.23%, 0.12% and 0.09% of the ICNIRP reference levels for each frequency band) respectively. The authors did not report the separate exposure levels for schools and homes. 

Another study [6] was based on temporal 24-h measurements of all the RF signals, including LTE, present in home and schools. Measurements were performed with spectral narrowband equipment. The maximal levels of exposure in the different frequency bands were found to be equal to 0.16 V/m (0.6% of the ICNIRP reference level) for broadcasting, 1.13 V/m for telecommunication (about 3% of the ICNIRP reference level), 3.10 V/m for DECT(about 3% of the ICNIRP reference level) and 1.70 V/m WiFi 2.4 GHz (about 2.8% of the ICNIRP reference level) in schools, and to 0.23 V/m for broadcasting (about 0.8% of the ICNIRP reference level), 0.12 V/m for telecommunications (about 0.32% of the ICNIRP reference level), 0.16 V/m for DECT (about 0.42% of the ICNIRP reference level) and 0.68 V/m for WiFi 2.4 GHz in homes (about 1% of the ICNIRP reference level). The largest maximal variations, defined as the ratio of the maximal momentary electric-field value to the minimum electric-field value over the considered time interval, were obtained for the cordless telephony (DECT) signals (10.6 dB) and for the WiFi 2.4 GHz signals (12.7 dB). Temporal variations of broadcasting signals and telecommunication signals were much lower. Thus, indoor sources exhibit the largest variations indoor and are the most critical for practical exposure assessment and comparison with existing guidelines.

A further comparison between the indoor exposure in different microenvironments can be found in a study by Birks et al. [2]. Personal RF-EMF exposure was measured in 529 children (ages 8–18 years) in Denmark, the Netherlands, Slovenia, Switzerland, and Spain by using personal portable exposure meters for a period of up to three days between 2014 and 2016 and repeated in a subsample of 28 children one year later. Results showed that median exposures in terms of power density were highest while children were outside (157.0 μW/m^2^, or 0.24 V/m, in terms of electric field strength, 0.86% of the ICNIRP reference level) or traveling (171.3 μW/m^2^, or 0.24 V/m, in terms of electric field strength, 0.9% of the ICNIRP reference level), and much lower when children were indoor, at home (33.0 μW/m^2^ or 0.1 V/m, in terms of electric field strength, 0.4% of the ICNIRP reference level) or at school (35.1 μW/m^2^ or 0.11 V/m, in terms of electric field strength, 0.4% of the ICNIRP reference level). The main contributions to the indoor exposure were found to be due to the downlink and broadcast sources.

A similar study by Frei et al. [75] examined the levels of exposure and the influence of different RF-EMF sources in a sample of 166 volunteers living in a Swiss city by using personal exposimeters. Participants carried an exposimeter for 1 week and completed an activity diary. Mean values were calculated using the robust regression on order statistics (ROS) method. The highest mean values of exposure were recorded for train journeys (1.16 mW/m^2^, 0.66 V/m), stays at the airport (0.74 mW/m^2^, 0.53 V/m) and rides on the tramway or bus (0.36 mW/m^2^, 0.37 V/m), while the smallest levels of exposure were measured in school buildings and kindergartens (0.02 mW/m^2^, 0.09 V/m), churches, cinemas and theatres (0.06 mW/m^2^, 0.15 V/m). In all locations, mobile telecommunication (up- and downlink) was the main source of exposure. Mobile phone base stations (downlink) were the most important contributor in churches (70.2%), in school buildings and kindergartens (56.0%), outdoor (52.6%) and at home (42.6%). In all other places, exposure was mainly due to mobile phones (uplink) (airport 95.2%; train 93.5%; cinema, etc., 82.8%; sports hall 79.1%; car 78.5%; tramway, bus 73.5%; hospital, doctor 69.0%; university, technical college 68.3%; restaurant, etc., 65.1%; shopping 60.2%; friends place, leisure residence 43.6%; workplace 29.0%). With respect to exposure in public transport, mobile phone uplink exposure was higher for persons using the mobile phone compared to people just exposed to the EMF generated by other user’s mobile phones (1.11 vs. 0.87 mW/m^2^ in trains and 0.27 vs. 0.23 mW/m^2^ in tramways and buses). Contributions from DECT were relevant at home (32.6%), at the workplace (24.1%). The contributions of FM radio and television (TV) broadcast transmitters were relatively small in all the indoor locations. Mobile phone uplink measurements during a call with a mobile phone were on average 4.87 mW/m^2^ (1.36 V/m, 3.5% of the ICNIRP reference level) and DECT measurements during a cordless phone call were equal to 2.98 mW/m^2^ (1.06 V/m, 2.7% of the ICNIRP reference level). Another study by the same authors [3] focused on the impact of personal phone use on personal exposimeter readings. The authors collected both personal exposimeter measurements during one week and spot measurements in the participants’ bedrooms. The mean personal exposure, obtained by averaging the results obtained during the whole week, was equal to 0.13 mW/m^2^ (0.22 V/m, 0.79% of the ICNIRP reference level), when measurements during personal phone calls were excluded and 0.15 mW/m^2^ (0.24 V/m, 0.85% of the ICNIRP reference level), when such measurements were included, while the mean level of exposure observed in the bedrooms of the involved subjects was equal to 0.11 mW/m^2^ (0.2 V/m, 0.73% of the ICNIRP reference level). Mean personal exposure in uplink frequency band calculated during the whole week of measurements increased by 0.038 mW/m^2^ (0.12 V/m) per hour of mobile phone use, while, as to the DECT frequency band, the mean exposure increased by 0.023 mW/m^2^ (0.09 V/m) per hour of cordless phone use. Exposure over all frequency bands along the whole week increased by 0.026 mW/m^2^ (0.1 V/m) per hour of mobile phone use and by 0.027 mW/m^2^ (0.1 V/m) per hour of cordless phone use. In case of mobile phone used by other people in the same environment, exposure to uplink increased by 0.023 mW/m^2^ (0.09 V/m) per hour of mobile phone use. The corresponding increase in the DECT band was 0.009 mW/m^2^ (0.06 V/m) per hour of cordless phone use. Total exposure calculated without personal phone use increased by 0.010 mW/m^2^ (0.06 V/m) per hour of mobile phone use and by 0.013 mW/m^2^ (0.07 V/m) per hour of cordless phone use.

A more recent study by Roser et al. [76] focused on the personal measurement of the RF-EMF exposure levels in Swiss adolescents. Even if the focus of the study was not the assessment of the exposure in indoor environments, the authors detailed the levels of exposure recorded in different indoor locations in which the involved subjects spent their time. The participants carried a portable RF-EMF measurement device measuring 13 frequency bands ranging from 470 to 3600 MHz for three consecutive days (with sampling interval equal to 4 s) and kept a time-activity diary. In total, 90 adolescents aged 13 to 17 years participated in the study conducted between May 2013 and April 2014. In addition, personal measurement values were combined with dose calculations for the use of wireless communication devices to quantify the contribution of various RF-EMF sources to the daily RF-EMF dose of adolescents. Results showed that the adolescents’ average total personal RF-EMF measurements were highest when spending time in public transport and cars (839.4 μW/m^2^, 0.56 V/m in terms of electric field strength, in cars, 676.3 μW/m^2^, 0.51 V/m in terms of electric field strength, in buses and 537.1 μW/m^2^, 0.45 V/m in terms of electric field strength, in trains), while the lowest measurements were measured at school (59.6 μW/m^2^, 0.15 V/m in terms of electric field strength) and at home (31.1 μW/m^2^, 0.11 V/m in terms of electric field strength). The main contribution for all the indoor locations was due to uplink signals, while the presence of WLAN at school and at home had little impact on the personal measurements (WLAN accounted for 3.5% of total personal measurements).

A very recent study by Gallastegi et al. [77] focused on the assessment of RF-EMF exposure in the Spanish children by spot and personal measurements in the locations where children tend to spend most of their time, i.e., homes, schools and parks, in order to identify which locations and sources contribute most to the level of exposure. 104 eight years children were involved: spot measurements were conducted in homes, schools and their play grounds and parks. At the same time, personal measurements were taken for a subsample of 50 children during 3 days. Results showed that median exposure levels ranged from 29.73 μW/m^2^ (0.11 V/m, 0.38% of the ICNIRP reference level) in children’s bedrooms to 200.10 μW/m^2^ (0.27 V/m, 0.88% of the ICNIRP reference level) in school playgrounds for spot measurements and were higher outdoors than indoors. The sources that contributed most to the exposure were FM radio, mobile phone downlink and Digital Video Broadcasting-Terrestrial, while indoor and personal sources contributed very little. Similar distribution was observed with personal measurements.

Two studies by Foster [17,78], carried out narrowband and broadband measurements in a high number of public indoor environments of different dimensions, including offices, hospitals, commercial locations, hotels and universities, for assessing the exposure due to the WiFi systems. The overall results for all the environments showed median time-averaged levels of exposure in the 70–3000 MHz band equal to 0.4 mV/m and median time-averaged RF power densities in the range of 1–10 μW/m^2^ at distances of about 1 m from a laptop when its Wi-Fi client was communicating with the network.

A study by Urbinello et al. [5] was focused on the characterization RF-EMF exposure levels and their temporal trends in typical everyday environments, including public transports, and indoor settings, by using micro-environmental measurements in Basel (Switzerland), Ghent and Brussels (Belgium). Measurements were collected every 4 s during 10–50 min per environment and measurement day. Linear temporal trends were analyzed by mixed linear regression models. The highest total RF-EMF exposure levels were found in public transports. In trains the exposure levels ranged between 0.83 V/m in Ghent (2.96% of the ICNIRP reference level) and 1.06 V/m in Brussels (3.79% of the ICNIRP reference level) and were higher compared to those of other environments, such as airports (maximum mean level equal to 0.54 V/m in Basel, 1.53% of the ICNIRP reference level), train station (maximum mean level equal to 0.57 V/m in Brussels, 2.03% of the ICNIRP reference level) and shopping centers (maximum mean level equal to 0.37 V/m in Bruxelles, 1.32% of the ICNIRP reference level). Mobile phones were the main exposure source in trains, whereas in other public transports and indoor environments mobile phone base stations (downlink exposure) had also a considerable impact on the exposure level. 

In a study by Joseph et al. [30] exposure to RF base stations for wireless technologies was assessed at 68 indoor locations, in Belgium, the Netherlands, and Sweden. The authors did not reported the levels of exposure observed in the different environment, but just the median and 95th percentiles considering the measures in all the considered locations. A total electric field with median value and 95th percentile equal to 0.28 V/m and 2.18 V/m, respectively, was found in indoor environments (equal to 1% and 7.8% of the ICNIRP reference level). The most influencing contribute was the exposure due to DECT, with exposure levels described by median value and 95th percentile equal to 0.12 V/m and 1.50 V/m (0.21% and 2.6% of the ICNIRP reference level), while contributions of LTE and WiMAX are on average 1%. Comparing the indoor exposure to the levels measured in the same areas, but outside buildings, thus in outdoor conditions, the authors found that 95th percentiles for total indoor and outdoor exposures did not differ much and were about 2.2 V/m. Higher total outdoor values of mobile telecommunication signals were obtained than at indoor locations, because only downlink exposure due to base stations was considered (discarding the contribution of uplink exposure due to mobile phones), which are outdoor sources and are dominant outdoor. Moreover, the propagation of base station signals into houses and buildings is subject to penetration losses.

Also in [79] the authors compared personal exposure obtained in different countries, i.e., Belgium and Australia, considering different microenvironments, included indoor places such as apartments, libraries, shopping centers and different types of transportation. Results showed that levels of exposure across the indoor environments were much lower than those outdoor, with the highest values in the library in Belgium (equal to 0.99 V/m) and the airport in Australia (equal to 0.27 V/m). 

In a study by Bolte et al. [21] the authors investigated the RF-EMF exposure level by using personal exposure measurements during daily activities of 98 volunteers living in The Netherlands. The mean exposure over 24 h, excluding own mobile phone use, was 0.180 mW/m^2^ (0.26 V/m, equal to 0.93% of the ICNIRP reference level). During daytime exposure was about the same, but during night it was about half, and in the evening it was about twice as high. The main contribution to environmental exposure (calling by participant not included) is from calling with mobile phones (37.5%), from cordless DECT phones and their docking stations (31.7%), and from the mobile phone base stations (12.7%). Instead of describing the detailed results by microenvironments, the authors reported the levels of exposure obtained during different daily activities of the subjects. As an example, the measurements recording while the subjects were waiting at a railway station showed the highest exposure, among all the considered activities, for the FM, TETRA, TV and the GSM and UMTS downlink bands. The exposure levels reordered at home showed mean power density equal to 0.159 mW/m^2^ (0.24 V/m, equal to 87% of the ICNIRP reference level), with different contributions of the different sources depending of the activities: for cooking, eating and sleeping the main contributor was WiFi (mean power density equal to 0.329, 0.04 and 0.03 mW/m^2^, respectively, equal to 0.35, 0.12 and 0.11 V/m, in terms of electric field strength), while for most of the other activities DECT gave the highest contribution (0.069 mW/m^2^, equal to 0.16 V/m in terms of electric field strength, corresponding to 0.28% of the ICNIRP reference level). For attending courses and for visiting church or museum the downlink bands were the main contributors. For shopping in supermarkets and in large stores and malls the uplink bands were the highest contributors, while for shopping in small shops the DECT band was the largest contributor. During public transport the highest exposure was from the GSM and DCS uplink bands (0.162 mW/m^2^ and 0.048 mW/m^2^, equal to 0.25 and 0.13 V/m in terms of electric field strength, corresponding to 0.65% and 0.23% of the ICNIRP reference level), but driving a car led to a higher exposure in case a passenger was calling (0.381 mW/m^2^, equal to 1 V/m in terms of electric field strength). Travelling by tram led to slightly higher exposure in the downlink bands than for other means of transport.

Finally, a comparison among the results of RF measurement campaigns in different urban areas across Europe using PEMs was performed by Joseph et al. [20]. The objective of this study was to compare personal radio frequency electromagnetic field exposure in different microenvironments between urban areas in five European countries, i.e., Belgium [80], Switzerland [25,75], Slovenia [81], Hungary [82], and the Netherlands [83]. In each of these countries large measurement studies using exposimeters were performed. Results showed that all mean values were well below the ICNIRP exposure guidelines. The highest exposure, mainly due to mobile phone handsets, was found in transportation vehicles (train and car/bus), with mean values of the power density in the range 0.3–1.4 mW/m^2^ (0.34–0.73 V/m, in terms of electric field strength), while the lowest exposure was found in urban homes, with mean values of the power density in the range 0.08–0.15 mW/m^2^ (0.17–0.24 V/m, in terms of electric field strength).

## 4. Discussion

The review of the research efforts towards assessing RF-EMF exposure in indoor environments in the last ten years reveals that comparing the results of exposure assessment in different studies is challenging and includes a lot of uncertainty. The various results show a limited representativeness, both because of the peculiarity of the experimental protocol used in each case and because often not all the context information is reported. 

Spot and long-term RF measurement campaigns described in different studies presented differences about the choice of the measurement sites in indoor environments. As defining a procedure that could be truly representative for population exposure is challenging and no standard procedure has been established so far, it was not possible to exclude that some of the studies reporting the highest or the lowest levels of exposure could have focused a priori on areas with enhanced exposure levels or with reduced exposure levels, respectively. Another important challenge for comparing the typical RF-EMF exposure values was the different kinds of devices used for exposure measurement, as different measurement settings may be chosen, with respect to frequency bands considered and to the sampling rate of the instrument. 

As to studies based on personal measurements, the use of different recruitment strategies, or the limited numbers of participants or microenvironments could make a direct comparison difficult. The results obtained in these studies could arise from few measurements in a high number of very different indoor environments, and could have been presented using different analysis methods, thus making very difficult to summarize general information about the levels of exposure. 

For both spot and long term measurements and personal measurements studies, the exposure levels reported in different publications where based on different averaging times (minutes, hours days). Moreover, different studies used different approaches for the handling of those values found to be below the detection limit of the measurement device. All these aspects could make results obtained in different studies difficult to compare.

Finally, results obtained by computational methods could be difficult to be summarized in order be representative for population exposure, as they are strongly depending on the exposure scenarios simulated in each specific study. Nevertheless, some overall exposure patterns can be derived to characterize the typical levels and contribution of different sources to the total RF-EMF exposure in various microenvironments.

Table 3 shows, a summary of the RF EMF exposure levels measured in different indoor locations obtained by spot and personal measurement and reported in literature. More specifically, the minimum and the maximum values among the mean and median values reported by the examined paper were reported in Table 3. No further arithmetic mean was applied, and all the studies for which the authors did not report median or mean values of thee exposure or did not detail the specific environment in which the value was observed were discarded. It should be notice that no normalization of averaging times was applied.

As a first common point among all the considered studies, all the results showed that in all considered environments the levels of exposure were well below the ICNIRP guidelines for general public exposure [43]. 

The highest maximum mean level of total exposure (i.e., the exposure due to the whole RF-EMF frequency band) was found in offices and was equal to 1.14 V/m. This value, reported by [70] as the total level recorded in offices in Belgium, was higher than all the mean/median values reported by all the other considered studies. Discarding this value, the maximum mean value for the total exposure in offices was found to be equal to 0.72 V/m. 

The second highest maximum mean value of total exposure was observed in public transport, equal to 0.97 V/m. This finding was highlighted also by single studies (see, e.g., [2,5,20,21]). This could be due to the concentration of people using mobile phones in public transport, usually higher than in other environments, leading to a higher probability of uplink communication and therefore higher exposures. Moreover, mobile phone use causes higher exposure for mobile scenarios (inside a train and car/bus) than for outdoor or stationary exposure due to changing environmental conditions, handovers from one base station to another, and higher required transmitted signal power from mobile phones to overcome penetration loss through (metallized) windows. This was confirmed by the maximum mean values for the uplink and downlink exposures reported in Table 3, equal to 0.96 and 0.85 V/m, respectively, and reported in literature for the transportation environments, and in particular for trains and cars.

Exposure at home was considered as particularly relevant as most people spend a great amount of time at home. In most of the analyzed studies, results showed that the levels of exposure in people homes were the lowest among all the observed environments [2,20,70,75], with mean values reported by the different studies in the range 0.13–0.81 V/m (see Table 3). This result could be due both to the presence of a lower number of indoor RF-EMF sources, compared to public environments such as offices, and to the fact that, at least for what concerns personal measurements, results about home exposure included nighttime observations, known to be characterized by lower levels of exposure compared to the daytime ones [20]. All the values reported in the study by Hardell et al. [59], related both to the broadband exposure level (equal to 0.81 V/m) and to the single contribution (e.g., the LTE downlink) were higher than all the mean/median values reported by all the other considered studies, probably due to the peculiarity of the apartment analyzed. When considering the other studies, analyzing the contribution of the different RF-EMF sources to the residential exposure, the maximum mean values was observed for the cordless phones (DECT) (equal to 0.35 V/m), whereas the WiFi was found to have lower contribution, with mean values ranging from 0.03 and 0.11 V/m. Our results about residential exposure levels are in line with those of the survey by Sagar et al. [4], who found mean values of exposure in the range 0.12–0.39 V/m. 

As to the public environments, the highest levels of exposure were found in offices, mainly due to the use of mobile phones (mean values of uplink exposure in the range 0.02–0.89 V/m) and to the use of DECT phones (mean values in the range 0.02–0.26 V/m). The outdoor base stations and the WiFi showed similar contribution to the levels of exposure, with mean values reported to be in the in the ranges 0.04–0.18 V/m and 0.03–0–19 V/m. 

The exposure in educational buildings, such as schools, universities, kindergartens, etc, were found to have exposure lower levels, with mean values of total exposure reported to be in the range 0.07–0.54 V/m, comparable to those observed in homes (see, e.g., [2]). In schools a very low contribution to the exposure level was due uplink or downlink mobile telecommunications, whereas the WiFi contributed to the exposure with mean levels in the range 0.01–0.29 V/m. Similar levels of exposure were observed in other public buildings, such as airports, railway stations, shopping centers, etc., with mean values of total exposure in the range 0.15–0.54 V/m (an higher level of exposure, equal to 0.99 V/m was found by Bhatt et al. [79] in the library in Ghent: the authors explained this finding with the position of the library, about 200 m from a nearby base station, which was exactly in line-of-sight).

About the contribution of different RF-EMF sources to the total level of exposure, even if slightly different patterns could be observed among the different indoor environments, results have to be interpreted with caution. As observed in the follow up study by Tomitsch et al. [19], the contribution of different sources to the measured in 2006, 2009 and 2012 varied with time, due on one side to the rapid evolution on one side of the telecommunication systems, and on the other side to the exponential growth in everyday life of use of technologies emitting RF-EMF. Therefore, results about the contribution of the different sources obtained in the examined studies have to be considered a time-dependent picture of the continuous evolving exposure to RF-EMF.

This is even more true if considering the forthcoming of 5G, with the expected huge increase of devices connecting by RF one each other. Even if all these technologies will be always more efficient in terms of power control and minimization of the emitted EMF, the assessment of the levels of exposure will still be crucial in order to ensure the safety of the new devices. In order to be able to take into account variability and uncertainty of the new exposure scenarios, stochastic approaches will be essential. These methods, being feasible to manage the uncertain aspects of the exposure scenarios, allow obtaining a stochastic and probabilistic description of levels of exposure in various environments, thus overcoming limitations of deterministic methods based only on measurements and computational methods, and giving results that could be truly representative for general population exposure.

## 5. Conclusions

In conclusion, this study provides a summary of the results obtained in the last ten years of research efforts focusing on the assessment of RF-EMF exposure and proving information specific to the levels of exposure in indoor environments. The results included a wide range of sources, both deployed outdoor or indoor the environment in which the exposure level was evaluated, a wide number of different environments, from offices and transportation, to private houses, and were obtained by different approaches, including spot and long-term measurements, personal measurements and computational methods. All the results showed that in all considered environments the levels of exposure were well below the ICNIRP guidelines for general public exposure [39], with the maximum mean levels of the exposure considering the whole RF-EMF frequency band in offices (1.14 V/m) and in public transports (0.97 V/m), and the lowest levels of exposure in homes and apartments, with mean values in the range 0.13–0.43 V/m. 

## Figures and Tables

**Table 1:** RF-EMF sources influencing the indoor exposure.

Location	RF-EMF Source	Communication Standard		Frequency (MHz)
Out	Base Station for radio	FM	Frequency Modulation	100
Out	Base Station for radio	DAB	Digital Audio Broadcasting	220
Out	Base Station for television	TETRA	Terrestrial Trunked Radio	390
Out	Base Station for television	Analogue TV	Analogue TV	174–223
Out	Base Station for television	DVB-T/TV	Digital Video Broadcasting–Terrestrial	470–830
Out	Base Station for television	UHF	Ultra-high frequency Television	470–860
Out	BS for mobile telecommunications	GSM900 DL	Global System for Mobile Communications	900
Out	BS for mobile telecommunications	GSM1800 DL	Global System for Mobile Communications	1800
Out	BS for mobile telecommunications	DCS1800 DL	Digital Communication System	1800
Out	Base station/Small cell	UMTS DL	Universal Mobile Telecommunications System	2100
Out	Base station/Small cell	LTE	Long Term Evolution	2600
In	Femtocell	UMTS DL	Universal Mobile Telecommunications System	2100
In	Femtocell	LTE DL	Long Term Evolution	2600
In	Access point	WIFI 2G	Wireless Local Area Networks	2400
In	Access point	WIFI 4G	Wireless Local Area Networks	2400
In	Access point	WiMAX	Worldwide Interoperability for Microwave Access	3500
In	Access point	WIFI 5G	Wireless Local Area Networks	5500
In	Mobile phone/Tablet	GSM900 UL	Global System for Mobile Communications	900
In	Mobile phone/Tablet	GSM1800 UL	Global System for Mobile Communications	1800
In	Mobile phone/Tablet	DCS1800 UL	Digital Communication System	1800
In	Mobile phone/Tablet	UMTS UL	Universal Mobile Telecommunications System	2100
In	Mobile phone/Tablet	LTE UL	Long Term Evolution	2600
In	Cordless phone	DECT	Digital enhanced cordless telecommunications	1880
In	Headphones, computer peripherals	Bluetooth		
In	Babyphones			

**Table 2:** Investigated indoor environments.

	Location	Population Density of the Area					Measurements		Simulation
		Urban	Suburban	Rural	Industrial	Not Specified	Spot/Long Term Measurement	Personal Measurement	
Public places	Schools	X	X	X		X	X	X	
	University					X	X	X	
	Kindergarden	X					X	X	
	Crèches	X	X	X			X	X	
	Offices	X	X	X			X	X	X
	Cinema	X						X	
	Church	X						X	
	Small shop						X	X	X
	Shopping center	X				X	X	X	
	Tourist visitor center		X				X		
	Railway station	X					X		
	Airport				X		X		
	Hotels	X	X				X		
	Health care facilities					X	X	X	
	Other Workplaces	X		X	X		X	X	
Private places	houses	X	X	X		X	X	X	
	bedroom	X	X	X			X	X	
	flat	X	X	X		X	X	X	X
Transportation	train						X	X	X
	bus						X	X	
	metro						X	X	
	car							X	X
	airplane								X
	public transport							X	
Other	indoor unspecified					X	X	X	X
	room					X	X		X
	elevator					X			X
	corridor					X			X

**Table 3:** Summary of the RF EMF exposure levels in different indoor locations: minimum and the maximum values among mean and median values reported in literature. When no paper reported information for specific locations and/or frequency band, the level was indicated as not available (n.a.). For each value the corresponding reference was reported. For the “total RF-EMF band” the values are reported also as percentage of the lowest reference level provided by ICNIRP guidelines for the corresponding frequency band (equal to 28 V/m) [39].

		Offices		Education Buildings		Residential Buildings		Other Public Buildings		Transportation									
										Generic		Bus		Train		Tram/Metro		Car/Taxi	
Frequency Band (MHz)		E (V/m) Mean Values		E (V/m) Mean Values		E (V/m) Mean Values		E (V/m) Mean Values		E (V/m) Mean Values		E (V/m) Mean Values		E (V/m) Mean Values		E (V/m) Mean Values		E (V/m) Mean Values	
		min	max	min	max	min	max	min	max	min	max	min	max	min	max	min	max	min	max
**Total RF-EMF**		**0.07**	**1.14**	**0.07**	**0.54**	**0.13**	**0.81**	**0.15**	**0.99**	**0.14**	**0.97**	**0.14**	**0.75**	**0.23**	**0.97**	**0.26**	**0.62**	**0.18**	**0.86**
% of ICNIRP ref. lev.		0.3%	4%	0.3%	1.9%	0.5%	2.9%	0.5%	3.5%	0.5%	3.5%	0.5%	2.7%	0.8%	3.5%	0.9%	2.3%	0.6%	3.1%
Reference		[79]	[70]	[52]	[70]	[56]	[59]	[75]	[79]	[62]	[5]	[62]	[81]	[62]	[5]	[2]	[79]	[62]	[61]
**Broadcasting**																			
FM	88–108	0.03	0.21	0.01	0.32	0.02	0.11	0.02	0.11	0.02	0.12	0.03	0.12	0.02	0.05	0.08	0.11	0.03	0.12
Reference		[21]	[70]	[52]	[70]	[83]	[80]	[21]	[21]	[20]	[80]	[62]	[80]	[20]	[5]	[21]	[62]	[25]	[20]
TV/DAB	174–223	0.01	0.77	0.01	0.12	0.02	0.07	0.00	0.04	0.02	0.07	0.02	0.05	0.02	0.03	0.03	0.03	0.02	0.07
Reference		[44]	[70]	[52]	[51]	[83]	[80]	[21]	[21]	[81]	[21]	[81]	[83]	[21]	[61]	[21]	[21]	[20]	[21]
Tetrapol	380–400	0.01	0.04	0.01	0.03	0.01	0.03	0.00	0.05	0.00	0.20	0.00	0.20	0.03	0.03	0.03	0.08	0.00	0.13
Reference		[70]	[44]	[18]	[54]	[70]	[82]	[21]	[21]	[20]	[61]	[20]	[61]	[61]	[61]	[21]	[61]	[21]	[61]
**Uplink Sources**																			
Total UL		0.02	0.89	n.a.	n.a.	0.07	0.23	0.03	0.23	0.03	0.96	0.07	0.66	0.17	0.96	0.04	0.41	0.03	0.66
Reference		[79]	[83]			[25]	[80]	[21]	[5]	[61]	[5]	[62]	[81]	[62]	[5]	[26]	[5]	[61]	[20]
LTE UL	800	n.a.	n.a.	0.01	0.04	n.a.	n.a.	n.a.	n.a.	n.a.	n.a.	n.a.	n.a.	n.a.	n.a.	n.a.	n.a.	n.a.	n.a.
Reference				[52]	[18]														
GSM UL	880–915	0.13	0.19	0.01	0.05	0.02	0.06	0.06	0.20	0.03	0.34	0.03	0.13	0.07	0.30	0.07	0.23	0.03	0.34
Reference		[44]	[21]	[52]	[18]	[26]	[21]	[21]	[21]	[26]	[21]	[26]	[21]	[26]	[21]	[26]	[21]	[26]	[21]
GSM/DCS UL	1710–1785	0.09	0.10	0.02	0.02	0.01	0.09	0.02	0.16	0.02	0.38	0.04	0.20	0.03	0.19	0.03	0.10	0.02	0.38
Reference		[44]	[21]	[18]	[54]	[26]	[21]	[21]	[21]	[26]	[21]	[26]	[21]	[26]	[21]	[26]	[21]	[26]	[21]
UMTS UL	1920–1980	0.00	0.03	0.01	0.01	0.02	0.04	n.a.	n.a.	0.02	0.03	0.03	0.03	0.03	0.08	0.03	0.04	0.02	0.03
Reference		[44]	[21]	[54]	[18]	[21]	[26]			[21]	[26]	[26]	[26]	[21]	[26]	[21]	[26]	[21]	[26]
LTE UL	2500–2570	n.a.	n.a.	0.01	0.01	n.a.	n.a.	n.a.	n.a.	n.a.	n.a.	n.a.	n.a.	n.a.	n.a.	n.a.	n.a.	n.a.	n.a.
Reference				[18]	[18]														
**Downlink Sources**																			
Total DL		0.04	0.18	n.a.	n.a.	0.04	0.23	0.04	0.94	0.01	0.85	0.01	0.39	0.07	0.33	0.23	0.53	0.12	0.85
Reference		[83]	[20]			[83]	[79]	[79]	[79]	[62]	[61]	[62]	[79]	[5]	[20]	[5]	[79]	[20]	[61]
LTE DL	700	n.a.	n.a.	0.01	0.01	0.35	1.1	n.a.	n.a.	n.a.	n.a.	n.a.	n.a.	n.a.	n.a.	n.a.	n.a.	n.a.	n.a.
Reference				[52]	[52]	[59]	[59]												
GSM DL	925–960	0.02	0.34	0.03	0.21	0.05	0.82	0.05	0.22	0.02	0.22	0.03	0.07	0.02	0.06	0.02	0.22	0.02	0.07
Reference		[26]	[70]	[18]	[70]	[21]	[59]	[21]	[21]	[26]	[63]	[26]	[21]	[26]	[21]	[26]	[63]	[26]	[21]
GSM/DCS DL	1805–1880	0.02	0.25	0.02	0.27	0.05	0.47	0.07	0.20	0.02	0.17	0.02	0.13	0.02	0.05	0.02	0.17	0.02	0.10
Reference		[26]	[70]	[52]	[70]	[70]	[59]	[21]	[21]	[26]	[21]	[26]	[21]	[26]	[21]	[26]	[21]	[26]	[21]
UMTS DL	2110–2170	0.00	0.28	0.02	0.13	0.02	0.7	0.03	0.08	0.03	0.05	0.03	0.04	0.03	0.04	0.04	0.05	0.03	0.04
Reference		[44]	[70]	[18]	[70]	[21]	[59]	[21]	[21]	[26]	[21]	[26]	[21]	[26]	[21]	[26]	[21]	[26]	[21]
LTE DL	2620–2690	n.a.	n.a.	0.02	0.02	0.2	2.1	n.a.	n.a.	n.a.	n.a.	n.a.	n.a.	n.a.	n.a.	n.a.	n.a.	n.a.	n.a.
Reference				[18]	[18]	[59]	[59]												
DECT	1880–1900	0.02	0.26	0.01	0.14	0.07	0.3	0.00	0.10	0.02	0.31	0.02	0.31	0.02	0.03	0.02	0.03	0.02	0.31
Reference		[83]	[44]	[52]	[70]	[70]	[82]	[21]	[21]	[21]	[20]	[26]	[81]	[21]	[20]	[21]	[26]	[20]	[20]
**WiFi**																			
Wifi/WLAN	2400–2500	0.03	0.19	0.01	0.29	0.03	0.11	0.02	0.03	0.01	0.07	0.01	0.04	0.02	0.05	0.03	0.05	0.01	0.07
Reference		[83]	[44]	[52]	[50]	[25]	[21]	[21]	[21]	[61]	[75]	[61]	[26]	[21]	[20]	[61]	[26]	[61]	[75]
WiMax	3400–3800	0.02	0.02	n.a.	n.a.	n.a.	n.a.	n.a.	n.a.	n.a.	n.a.	n.a.	n.a.	n.a.	n.a.	n.a.	n.a.	n.a.	n.a.
Reference		[44]	[44]																
Wifi5G	5150–5850	0.13	0.25	0.00	0.03	n.a.	n.a.	n.a.	n.a.	n.a.	n.a.	n.a.	n.a.	n.a.	n.a.	n.a.	n.a.	n.a.	n.a.
Reference		[44]	[36]	[54]	[18]														
